# Evaluation of Early and Late High School Student Science Research and Mentorship Programs: Virtual Gateway to Science Curricula and Mentorship During the COVID-19 Pandemic

**DOI:** 10.15695/jstem/v6i1.07

**Published:** 2023-04-25

**Authors:** Lauren Wozniak, Alexis Guzman, Sheila McLaughlin, Bonnie Halpern-Felsher

**Affiliations:** Division of Adolescent Medicine, Department of Pediatrics, Stanford University, Palo Alto, CA

**Keywords:** STEM, Virtual Programs, Mentorship, High School, Pandemic, Underrepresented Youth

## Abstract

People from racial and ethnic minoritized groups, those with disabilities, and those from low-income backgrounds are underrepresented in biomedical careers. Increasing diversity in the biomedical workforce, particularly health care providers, is imperative to address the disparities faced by minoritized patients. The COVID-19 pandemic highlighted disparities experienced by minoritized populations and emphasized the need for a more diverse biomedical workforce. Science internship, mentorship, and research programs, which have historically been conducted in person, have been shown to increase interest in biomedical fields for minoritized students. During the pandemic, many science internship programs pivoted to virtual programming. This evaluation focuses on two such programs for both early and late high school students and evaluates change in scientific identity and scientific tasks pre- and post-program. Additionally, early high school students were interviewed to obtain more in-depth information on the program experiences and effects. Early and late high school students reported increased scientific identity and comfort with scientific tasks compared pre- to post-program in several domains. Desire to pursue biomedical careers was maintained pre- to post-program for both groups. These results highlight the importance and acceptance of developing curricula for online platforms to help boost interest in biomedical fields and desire for biomedical careers.

## INTRODUCTION

People from racial and ethnic minoritized groups and those from low-income backgrounds are underrepresented in higher education and science-related careers (De Brey et al., 2019). Adolescents from these groups are more likely to come from families with low educational attainment, attend schools with more limited academic opportunities and resources, and live in areas with little exposure to role models in the biomedical sciences (De Brey et al., 2019; Shin et al., 2016). Adolescents from these minoritized and low-income groups are less likely to attend and complete college, obtain a science degree, or pursue a science-related career (Nadeem, 2021). However, having a healthcare and scientific workforce that includes people from low income and minoritized groups is critical.

The demographics of the United States are changing, with the US Census Bureau predicting that approximately one-third of the population will be a race other than white by the year 2060. Those who identify as “two or more races” are the fastest growing group in the US and the non-Hispanic White population will decrease by almost 19 million individuals by 2060, with non-Hispanic Whites expected to become the minority group in the US by 2045 (Vespa et al., 2020). With this shift, the racial and ethnic makeup of the biomedical workforce must change to match the composition of the US population. It has been demonstrated that patients from minoritized groups are more likely to experience health disparities including reduced life expectancy, decreased quality of life, and fewer economic opportunities (National Academies of Sciences, 2017). However, patients who receive care from clinicians of similar backgrounds experience less bias (Hagiwara et al., 2017), which may help to reduce health disparities. As such, providing opportunities to expose adolescents from minoritized and low-income backgrounds to science and health-related fields is critical to encourage higher education and careers in these fields. Science pipeline programs have played an important role in exposing underrepresented students to science-related careers, professionals who provide mentorship, and academic enrichment opportunities (Winkleby et al., 2015; Winkleby and Ned, 2010).

The COVID-19 pandemic has been one of the largest public health emergencies of the last century, affecting millions of people worldwide and placing significant strain on the healthcare workforce and public health infrastructure. In the United States, the pandemic widened racial, ethnic, and socioeconomic disparities based on access to testing, vaccine uptake, the incidence of infection, and mortality rates, which were often worse among minoritized populations and those from low-income backgrounds (Bambra et al., 2020; Lieberman-Cribbin et al., 2020; Nguyen et al., 2022; Tai et al., 2021). The challenges encountered during the pandemic not only highlighted the importance of the need for a diverse healthcare and scientific workforce but also exacerbated educational disparities experienced by adolescents from disadvantaged backgrounds (Haderlein et al., 2021). Lockdowns to reduce infection rates created widespread isolation and left many young people with the inability to go to school, learn directly from their teachers, or participate in traditional in-person academic enrichment programs.

During the COVID-19 pandemic, universities that hosted in-person science pipeline programs for underrepresented students pivoted to virtual programs to continue to reach students. Programs have included participation from students in grade school to the college level, have lasted from weeks to months, and have taken place during the school year or summertime. The content of the different programs has included education on a variety of science topics, including workshops to improve writing and reading skills, career workshops, the conduct of scientific experiments and research, mentorship opportunities, and support sessions to learn stress management skills.

While there are successful models for transitioning from an in-person to virtual platform for science education programs, (Bradley et al., 2021),(Stainbrook, 2022), evaluation of the success and impact of these programs is ongoing. Thus far, evaluations have shown some promising results of these virtual science programs. Virtual programs conducted to date have been acceptable to students, shown to increase interest in and knowledge of science, technology, engineering, and math (STEM) fields, and have provided students with academic preparation including research skills (Chang et al., 2021; Frost et al., 2021; Fung et al., 2021; Karara et al., 2021; Nazario-Leary et al., 2021; Qua et al., 2021; Scott Price et al., 2020). The program evaluations often focused on the acceptability and feasibility of the program and on the effectiveness of the program on increasing interest in pursuing a science-related career. Most of this information has been obtained through program evaluations using pre- and post-survey data and from students in one regional area. Studies using qualitative data to provide a deeper understanding of students’ perspective of these virtual programs have been more limited.

The goals of this evaluation were to assess two high school science programs with larger catchment areas. We utilized mixed methods, both pre- and post-program surveys and qualitative interviews, to determine whether two high school virtual biomedical and health science-focused programs, one for 9th and 10th grade students and one for 11th and 12th grade students, were acceptable, changed students’ interest and confidence in pursuing a science-related career, and increased their sense of identity and belonging in the scientific community. We also examined students’ perceptions of the programs taught within a virtual setting.

## METHODS

### Program Overview.

We administered and evaluated two NIH-funded high school programs which were part of the NIH/NIDDK-funded Short-Term Research Experience Program to Unlock Potential (STEP-UP). The two programs, the Kickstarter program for 9th and 10th graders (early high school program) and the High School Program in Biomedical and Health Sciences for 11th and 12th graders and recent high school graduates (late high school program), seek to expose underrepresented students from various geographic locations to the scientific community, enhance scientific identity, promote interest in STEM fields, and provide research experiences. The ultimate goal of both programs is to increase the number of high school students who are committed to and well-positioned for pursuing careers in the sciences. The programs provide opportunities for high school students to develop critical thinking and educational skills within a supportive environment in which they have sustained relationships with adults (university faculty, medical and graduate students, and postdoctoral fellows) as well as peers in the program. Priority is given to recruiting and accepting students from populations less represented in the biomedical and health sciences. Our programs were developed on sound research showing that structured out-of-school science programs can stimulate science-specific interests among students, positively influence academic achievement, and expand participants’ sense of future science career options (Bell et al., 2009; Halpern-Felsher and McLaughlin, 2016; Rivers et al., 2020). In addition to focusing on improved academic skills, our programs strive to facilitate the development of positive attitudes toward science, scientific skills, and positive relationships with peers and mentors. This is particularly important for minoritized students who often arrive at these programs with lower levels of confidence with math and science than do white youth (Bell et al., 2009; Halpern-Felsher and McLaughlin, 2016; Rivers et al., 2020).

In the early high school program, students conducted research remotely during the academic year, from October 2020 through May 2021. Students and program staff met after school once a week via Zoom for didactics and to discuss the student research projects. Students then worked in small groups with their science high school teachers to conduct their pilot projects. The two teachers participating in the early high school program were recruited based on previous involvement with helping to recruit students for the late high school program. Both were science teachers who had expressed interest in participating in the program and mentoring students in conducting research projects.

In the late high school summer internship program, each student was paired with an adult scientific mentor, usually a professor, postdoctoral fellow, and/or graduate student, who enabled students to obtain first-hand virtual experience in a scientific environment. Scientist mentors for the late high school program were generally faculty members recruited from universities proximal to the students’ homes. Many of these mentors had participated in our high school internship program in the past. Scientist mentors were expected to work with the students over the summer to develop a project and/or research question and to mentor the students in addressing that question over the summer. Mentors often had a graduate or medical student or fellow in the lab as the direct, day-to-day mentor for the late high school students. Students were provided information on their mentor’s lab research and were coached on reading and reviewing literature relevant to the lab. Students then met with their mentors to discuss possible projects that were feasible to conduct online and in a short amount of time. Some example projects include literature reviews, analyzing existing large-scale datasets such as the National Youth Tobacco Survey, or analyzing specific lab-based datasets. Students then spent 8–10 weeks working in research over the summer virtually, with the exception of one student who was in-person, and they attended seminars three times a week, each for about 2 hours, via Zoom. For both the early and late programs, the didactic topics included writing a scientific abstract, professional development, research methods and statistics, giving scientific presentations, and ethics in research. Sessions also included detailed instructions and information about getting into and funding college, journal clubs, career development panels, diversity in medicine, community engagement, advocacy and policy, and giving an “elevator pitch” about their background, experiences, and research projects. Students were given the opportunity to present their research findings among their peers and mentors. Oral presentations helped students gain confidence in public speaking regarding scientific topics and allowed peers the opportunity to provide feedback. The ongoing program meetings not only provided various training opportunities but were also meant to foster an atmosphere where students could network and learn from each other.

Program staff checked in frequently with both the scientist mentors and teachers to assess the programs, assess if individual students were on track, and if there were any questions or concerns. Program staff worked with the mentors and teachers to address any of these issues or questions. Importantly, support for students continued after completion of the program. Program staff and leadership continued to work with the students throughout the academic year, mostly for later high school students, to help them apply to college, review college essays, write letters of recommendation, and provide other support that students might request. Additionally, some students continued with their projects throughout the year and into the following summer.

### Student Selection and Demographics of Participants.

The late high school program recruits and selects students in either 11th or 12th grade who are typically underrepresented in the STEM fields. Due to the COVID-19 pandemic a small number of students whom had been accepted to the program while in 12th grade were enrolled in college after the program was resumed. There are four coordinating centers that, along with the NIH, work to recruit students throughout the United States. Recruitment is through word-of-mouth from alumni, social media, educational conferences, and contact with high schools throughout the US. Eligible students had at least a 3.0 GPA, completed an online application including a personal statement, and submitted letters of recommendation and academic transcripts.

The early high school program recruited a total of 10 students from partnering schools in identified underrepresented districts. All participants in the early high school program were 9th or 10th graders, had at least a 3.0 GPA, demonstrated interest in the sciences, submitted letters of recommendation, and completed an application essay. Partnering schools and science teacher mentors helped to recruit students and promote interest.

Students in the late high school program received $2500 for participation in the program. Students in the early high school program received $1000 for participation and an additional $25 for participating in the qualitative interview. There was no additional compensation provided for completion of the pre- and post-survey for either group.

### Data Collection and Analysis.

Prior to participation in the program, students from both the early and late high school groups completed a pre-survey querying about student and family demographic information, including educational attainment for students’ parents or guardians (Table 1). In addition, there were 38 items addressing self-perceived competency in scientific skills and scientific identity. Students were asked to respond to each item on a 5-point Likert-type scale, from strongly disagree (5) to strongly agree (1). In our analysis, we reverse coded this scale to correspond to (1) strongly disagree and (5) strongly agree to allow for ease of interpretation. In addition, there were seven items concerning interest in pursuing higher science education and career goals, which were answered using a 4-point Likert-type scale from not interested (1) to very interested (4), as well as a not sure response (5). This survey was repeated after program completion for both groups and additional questions including desire to pursue additional STEM programming and overall program evaluation were included in the post-program survey. Of the 25 late high school students, 22 responded to the survey and eight of the 10 for the early high school group responded. Related samples Wilcoxon Signed Rank Tests were conducted using SPSS version 28.0.1.1 for participants in both programs to assess differences in the student ratings of scientific identity, ability to complete scientific tasks, and desire to participate in scientific education and careers preversus post-program.

Of the 10 initially enrolled students, eight students completed the early high school program. The eight students and two teachers who completed the early high school program also completed a 1:1 qualitative interview post-program with either authors LW or AG. All sessions were recorded virtually on the Zoom platform with the knowledge and consent of the participants. Assent from the students and consent from the teachers was obtained prior to the interview (since this was considered program evaluation, Stanford University did not require formal parental consent or IRB approval).

Transcripts from the Zoom platform were reviewed by LW and AG for accuracy and compared to the original recording and edited to reflect what was said by the participants. The transcripts of all interviews were then individually coded by LW and AG utilizing NVIVO software. Any discrepancies in coding were resolved through discussion between LW, AG, and BHF. The qualitative interview addressed the students’ and teachers’ thoughts about the program overall, feelings around COVID-19 and the impact on learning, virtual versus in person programming, the research projects, community involvement, and mentorship.

## RESULTS

### Demographic Data.

We had 10 students in the early high school program, of whom eight were in 10th grade and two were in 9th grade; seven were female and three were male. The mean age was 14.0. Six students were Black, one was Asian, one was White, one Hispanic, and one American Indian/Alaskan. Half of the students had parents who had not attended college (Table 1).

There were 25 enrolled students in the late high school program, of whom 11 had completed 11th grade, four had completed 12th grade, and 10 were in college. Thirteen identified as female and 12 as male, with a mean age of 17.4 years. Ten students were Black, eight Hispanic, five Asian, six White, and one identified as other for race and ethnicity. Nine of these students had parents who did not attend college.

### Survey Data.

Pre- and post-surveys evaluating students’ self-reported confidence in scientific skills and identity were conducted. Significant comparisons are noted in Table 2 and Table 3 (P<0.05) and described below.

In comparing responses between the pre- and post-program surveys, results showed that after early high school program participation, students were more likely to report they were able to write research reports (p = 0.023), felt part of a scientific community (p = 0.034), and felt able to get support from professionals about interests in science (p = 0.038). Early high school program students also reported an increase in enjoyment in working on science problems (p = 0.02), an increased understanding of the ethical considerations in conducting research (p = 0.046) and increased knowledge of how to communicate clearly and professionally in the research environment (p = 0.038). Following participation in the program, early high school students also were more likely to see themselves as a scientist (p = 0.038) and think that a career as a scientist includes participating in the broader scientific community (p = 0.038) (Table 3).

For the late high school students, in comparing responses between the pre- and post- program surveys following participation in the program, results showed that late high school students reported greater confidence in ability to contribute to science (p = 0.002), comfort working collaboratively with others (p = 0.034), ability to analyze data for patterns (p = 0.032), ability to formulate a research question (p = 0.002), comfort giving oral presentations (p = <0.001), ability to write research reports (p = <0.001), feeling like a scientist and a part of the scientific community (p < 0.001), ability to get support from professionals about interest in science (p = 0.01), confidence in ability to succeed in a science major (p = 0.003), and confidence in ability to succeed in a science – related career (p = 0.012) (Table 2). Additionally, following the program, late high school students reported greater familiarity with tasks performed by a scientist (p = 0.013); belief that a career as a scientist would be enjoyable (0.003); positive impression of the scientist career (p=0.001); enjoyment working on science problems (p = 0.011); feeling that while working on a science problem [they] sometimes don’t notice time passing (p = 0.008); a good understanding of the ethical considerations of conducting research (p >0.001); knowledge of conducting research responsibly and safely (p = 0.019); knowledge of communicating clearly and professionally in the research environment (p = 0.005); confidence in time management skills (p = 0.012); thinking of [themselves] as a scientist (p = 0.002); and seeing [themselves] as a scientist (p = 0.003) (Table 3).

Data were also collected regarding participant interest in pursuing future scientific opportunities and scientific careers (Table 4). Responses of “unsure” were not included in the analysis. There were no differences pre- to post-program for any item for either early or late high school students. Pre-program data demonstrated high interest for late high school students for pursuing more research opportunities in high school, pursuing an undergraduate degree, obtaining advanced degrees, and pursuing a career in research (mean score >3 with a score of 3 defined as “moderately interested”). This level of interest was preserved on post-survey responses for late high school students. Early high school participant responses demonstrated similar trends.

### Qualitative Findings.

Five themes emerged from our interviews with early high school students and their two teachers regarding the virtual program: 1) career and future directions for students, 2) science identity and science knowledge, 3) diversity and mentorship, 4) the impact of COVID-19, and 5) program design / feedback. Each of the themes is described in detail below with supportive illustrative quotes in Table 5. Late high school students did not complete a qualitative 1:1 interview, but did leave qualitative feedback on post-program surveys, which is detailed below in Table 6.

#### Career/Future Directions.

Many participants reported a desire to incorporate STEM into future careers and several participants expressed a desire to pursue the medical field. Some participants discussed a desire to pursue law or business, but also stated that they felt that they would incorporate STEM ideas into future careers. Additionally, some participants mentioned that because of the program they thought that adding research to a future career would be of interest.

#### Science Identity/Science Knowledge.

Participants were asked to describe their definition of a scientist and discuss if their opinion on what a scientist is changed during the program. Many participants expressed a change in their opinion on the role of science and science careers after completing the program in comparison to before the program. For instance, several students mentioned that they had previously seen a career in science as most similar to typical bench research. Post-program students reported that they saw a career in science as one that focused on addressing multitudes of questions beyond those addressed solely in a lab setting. Participants were also asked about whether they personally felt like scientists and whether they felt like a part of the scientific community. Some participants expressed that while conducting research projects in this program, they did feel ‘like a scientist’ and the ability to connect to mentors in the STEM field made them feel part of the scientific community. One participant discussed that they felt like a scientist while conducting research, but after completion of the program they no longer felt like a scientist because they were not actively working on a research project.

#### Diversity and Mentorship.

Participants were asked to describe previous experiences with mentorship in the STEM fields. Many participants expressed that they did not previously have exposure to mentors in scientific careers and felt that it was helpful to hear from people in STEM careers in the program. Participants also reported feeling that it was helpful to have mentors in the program who had similar racial and ethnic backgrounds.

#### COVID-19 Impact/Virtual Programming.

Participants reported that if COVID-19 wasn’t happening that they would have enjoyed in-person meetings, but also reported that the virtual meetings were fun and engaging. Some participants also discussed how the program served as a distraction from the COVID-19 pandemic. There were varying opinions about in-person versus virtual programming. While many participants reported that they would have preferred for the program to be in person, the majority also recognized the benefit of virtual programming, including the ability to meet students from other states. Some participants also appreciated the flexibility offered by virtual programming.

#### Program Design/Feedback.

Participants were asked to describe the curricular aspects of the program, their expectations for the program, the research topic addressed, and the time dedicated to the program. Overall, they reported that the program had met their expectations for learning and found it exciting to participate in research. Participants also expressed that the time dedicated to the program was appropriate overall. Some participants mentioned that a longer time frame for programming might have been helpful to work on their research project. Teacher participants also discussed that they spent extra time outside of formal programming to work with students on research project design and completion and found that the year-long curriculum was helpful to work through the research projects. Participants were also asked about any difficulties or challenges they had experienced in the program. Many discussed challenges with working in groups virtually and found student engagement difficult with many participating off camera.

Late high school students did not participate in 1:1 interviews post-program to provide feedback but did offer qualitative written data on post-program surveys specifically commenting on what they enjoyed about the program and what they would like to see changed in the program. Similar to early high school students, late high school students reported that the program was a valuable use of time and important for their development as scientists and researchers. The mentorship exposure was also important to the students. However, many late high school students also reported that the virtual setting of the program made it challenging to promote social interaction and collaboration. Students stated that they would have enjoyed being in a lab setting in-person. Select illustrative comments from late high school students are shown below (Table 6). Of note, though no formal evaluation was conducted for late high school scientist mentors, informal weekly email check-ins with program staff and mentors demonstrated very positive feedback on the program.

## DISCUSSION

Increasing underrepresented and minoritized student interest in the STEM fields is critical as the population of the US grows and changes. High school science programs seek to expose underrepresented students from different geographic locations to the STEM fields and promote interest in pursuing biomedical careers. In our two high school programs, for early and late high school students, students work with research mentors and peers to develop a research question and project. Students also connect with a variety of mentors in STEM fields providing valuable exposure to different career paths. The availability of mentors from different fields including traditional medical settings, biomedical research, and public health specialists could have contributed to students reporting that they had a wider view of the scientist role in the post survey. Many students commented in interviews that they had previously seen scientists as something closer to one who conducts bench research, but post-program many students remarked that they learned that science expands beyond more traditional bench work.

Previously it was common for STEM programs to be delivered in person to allow for hands-on participation in research projects and socialization with peer and mentor groups. However, with the COVID-19 pandemic, programs were forced to transition to virtual models. Our early and late high school programs were also delivered via a virtual model. The challenge with virtual models has been preserving rigorous programming to ensure that critical knowledge and skills are gained from the experience. As a benefit, however, virtual programming offers a way to reach a wider geographic area of students who might not have had exposure to STEM programs from higher education institutions in the past.

This mixed-methods evaluation demonstrated the impact of a virtual science curricula for students from different geographic areas. With this virtual programming, students in both the early and late high school programs reported more confidence in scientific and research skills and also reported increase in scientific identity. Specifically, students commented on the benefit of formulating research questions and conducting data collection in qualitative interviews. Many reported that they felt more comfortable conducting research and anticipated that research would be an important part of future careers. There are some differences between the early and late high school students in the results. Specifically, late high school students seem to have more statistically significant increases in scientific identity and skills compared to early high school students. This could be due to several factors including larger sample size of the late high school group or the age and exposure to STEM experiences for late high school students. Early and late high school programs were different in timing as well, with early high school programming happening during the academic year and late high school programming during the summer. This could also contribute to differences in results since summer students may have been able to fully focus on their research. Some early high school students commented on the fact that they found it challenging, at times, to focus on both school and the STEM program. In the future, offering summer programming to early high school students and comparing impact on scientific identity and skills would be helpful to distinguish the preferred method for offering virtual programs.

Overall our results demonstrate that it is reasonable and possible to deliver robust STEM programming virtually and is consistent with prior reports (Qua et al., 2021; Stainbrook, 2022). However, particularly with late high school students, many reported that they would have liked learning scientific skills in a traditional hands-on lab setting. Future programs could explore how to provide exposure to traditional lab settings and skills while maintaining the benefits of virtual programming. It is important to recognize that traditional lab settings can introduce high costs to programs and limit student participation to those who are in close proximity or have the means to travel for the experience. Rather, future programs could emphasize that the traditional research skills of developing questions and problem statements, developing a research plan, conducting research, and completing data analysis are the critical skills that must be learned for a career in science. These skills translate well to virtual programming as supported by our data.

Despite the challenges of the COVID-19 pandemic, students were able to fully participate in the virtual sessions and were exposed to students from various geographic regions of the country. Many students expressed the desire for in-person programming, but also reported that virtual programming was acceptable and sometimes beneficial. Virtual programming offered increased flexibility for scheduling and exposure to people from different areas of the country, which students enjoyed. Some students commented on peer engagement during the sessions and specifically remarked that others might have their cameras off during the program. To help with student engagement and virtual etiquette, program leadership discussed virtual Zoom etiquette at the beginning of the program as well as periodically during the program. Students were consistently asked to have on their cameras and to participate by answering questions and interacting either verbally or at least utilizing the chat function. These reminders were useful, but program staff and teachers also commented that some students had unstable internet access, might live in small apartments, would have other family including younger siblings at home, or might have other disruptions which made having cameras on or interacting difficult. Future programs should utilize virtual programming when possible, but also must recognize that these challenges exist for students and should consider identifying or developing creative ways to improve and assess engagement.

All early high school students stated that they found this program worthwhile on quantitative analysis from post-survey data. The majority of students from both the early and late high school groups stated that they would participate again if the program was offered in the coming year and that they were likely to recommend the program to other students, which demonstrates acceptability of virtual STEM programming. Additionally, all early high school students reported a desire to pursue additional STEM programming in high school.

We have limited feedback from scientist mentors in the late high school program, but informal weekly email check-ins with program staff demonstrated positive review for the program. Teachers in the early high school program provided qualitative feedback that they found the program to be a positive experience for their students. Teachers also reported some additional time was needed outside of online sessions to help students complete their projects and to provide mentorship. More formalized feedback on mentor and teacher impressions of the program and time spent outside of dedicated sessions would be helpful for program development in the future.

### Limitations.

There are several limitations to this evaluation. All students who participated in virtual programming had access to internet and computers, which may be challenging for students from other areas. Furthermore, students who participated in these programs did so by applying after they were recommended to do so by their teachers and were generally high achieving students interested in STEM. Additionally, this program evaluation is based on short-term data collected from the early and late high school students and therefore cannot comment on the longevity of the impacts from this programming. Finally, due to the COVID-19 pandemic a small number of late high school students had to delay participation in the program and were already accepted to college by the time they enrolled in the program.

### Implications.

Despite the limitations noted, this evaluation allowed for an in-depth assessment of how students’ scientific identity and perception of their skills changed via an early high school and late high school extracurricular STEM program. Our mixed-methods analysis for early high school students also allowed for an in-depth assessment on the acceptability of virtual programming and provided robust qualitative data from student and teacher participants. Our results demonstrate that virtual programming allows for students from different geographic regions to participate in rigorous STEM extracurricular curricula and that, in general, youth find this modality an acceptable alternative to in-person programming. In addition, they reported increase confidence in scientific skills, knowledge, and identity. Virtual curricula are promising because they allow students who typically do not have access to programs providing research experience or mentorship experience an opportunity to engage in STEM (Johnson et al., 2020). Students voiced that they would have preferred in person programming over virtual, but also recognized the benefits of virtual programming as described above: increased access to remote academic institutions and high-quality mentorship. Despite the reported preference, students also reported that they would be likely to recommend the program to their peers and consider additional STEM programs if they were to be offered again in the future. For late high school students, they voiced that they would have liked more opportunity to socialize with peers and form connections with peers and mentors. Several students also expressed a desire to work in a physical lab.

Ultimately, STEM programs should be designed to increase diversity in STEM degree programs and career fields. There is some evidence that structured research and mentorship programs that increase scientific identity and skills contribute to underrepresented students participating in STEM fields or obtaining STEM degrees in the long term (Chemers et al., 2011; Estrada et al., 2018; Gibson and Chase, 2002; Salto et al., 2014). However, this study is reflective of virtual programming in the short-term. Long-term impacts of virtual programming and mentorship will need to be further evaluated as programs continue to move to more virtual models.

Future programs and studies should examine how to integrate important aspects of in-person programming, such as socialization and networking that are critical to successful careers in biomedical fields, into virtual models while maintaining rigorous educational curriculum. Virtual programs for underrepresented and minoritized youth can increase access to otherwise more difficult to attend programs. Virtual programs should be continued even when in-person activities are resumed and long-term impacts of these programs should be assessed.

## Figures and Tables

**Table 1.** Demographic information for students enrolled in the early and late high school programs.

	Late High School Program	Early High School Program
**Gender**		
Female	13	7
Male	12	3
**Mean Age**	17.4 years	14.0 years
**Race / Ethnicity**		
Black	10	6
Hispanic	8	1
Asian	5	1
White	6	1
Native American	0	1
Other	1	0
Preferred not to answer	0	1
**Grade During Program**		
In 9th grade during the program	N/A	2
In 10th grade during the program	N/A	8
Completed 11th Grade	11	N/A
Completed 12th grade	4	N/A
In College	10	N/A
**Family Educational Attainment**		
Parents not attended college	9	5

**Table 2.** Reported Science Identity for Early and Late High School Students Pre- versus Post-Program Survey.

	Early High School Program			Late High School Program		
	Pre-survey mean	Post-survey mean	P-value	Pre-survey mean	Post-survey mean	P-value
Rate how much you agree/ disagree with each of the following:						
I have confidence in my ability to contribute to science	3.8	4.3	0.18	4.095	4.714	**0.002**
I am comfortable working collaboratively with others	4.1	4.3	0.564	4.381	4.667	**0.034**
I am able to work independently	4.1	4.4	0.414	4.524	4.81	0.058
I am able to analyze data for patterns	3.6	4.3	0.129	4.095	4.571	**0.032**
I am able to problem solve	3.9	4.3	0.18	4.134	4.429	0.083
I am able to formulate a research question	3.4	4.4	0.059	3.857	4.667	**0.002**
I am comfortable giving oral presentations	3.3	4	0.062	3.467	4.517	**< 0.001**
I am able to write research reports	3.1	4.1	**0.023**	3.762	4.667	**< 0.001**
I am able to create a scientific poster	3.8	4.3	0.234	3.619	3.952	0.182
I am able to conduct scientific experiments	3.6	4.4	0.063	3.81	4.238	0.083
I am able to keep a detailed lab notebook	3.5	4	0.194	4.0	4.142	0.366
I am able to manage my time	3.6	3.9	0.739	4.095	4.333	0.132
I feel like a scientist	3.3	4	0.096	3.19	4.333	**< 0.001**
I feel part of a scientific community	3.4	4.1	**0.034**	3.286	4.524	**< 0.001**
I am able to get support from professionals about my interests in science	3.4	4.3	**0.038**	3.905	4.714	**0.01**
I am confident in my ability to succeed in a science major	3.4	4.1	0.109	4.048	4.667	**0.003**
I am confident in my ability to get into college	4.3	4.5	0.317	4.619	4.762	0.317
I am confident in my ability to succeed in a science - related career	3.6	4.4	0.059	4.143	4.619	**0.012**
**OVERALL**	3.617	4.21	**0.017**	3.947	4.521	**<0.001**

**Table 3.** Perceptions of Scientific Tasks for early and late high school students on the Pre- and Post-Program Survey.

	Early High School Program			Late High School Program		
	Pre-survey mean	Post-survey mean	P-value	Pre-survey mean	Post-survey mean	P-Value
Indicate how much you agree or disagree with these statements:						
I am familiar with the tasks performed by a scientist	3.8	3.8	1.0	3.524	4.19	**0.013**
I believe a career as a scientist would be enjoyable	4.3	4.3	1.0	4.048	4.619	**0.003**
I have a positive general impression of the scientist career	4.1	4.4	0.317	4.19	4.762	**0.001**
It is important to me to be a good scientist	4	4.3	0.589	4.476	4.762	0.058
I enjoy working on science problems	3.4	4.3	**0.02**	4.286	4.667	**0.011**
Science is one of the things that is important to me personally	3.6	3.6	1.0	4.381	4.571	0.102
I would even give up some of my spare time to learn new topics in science	3.1	3.4	0.157	4.381	4.429	0.763
While working on a science problem it sometimes happens that I don’t notice time passing	3.5	3.8	0.48	3.667	4.286	**0.008**
I have a good understanding of the ethical considerations in conducting research	3.8	4.3	**0.046**	3.952	4.762	**<0.001**
I know how to conduct research responsibly and safely	3.6	4.1	0.102	4.0	4.524	**0.019**
I know how to communicate clearly and professionally in the research environment	3.4	4.3	**0.038**	3.81	4.571	**0.005**
I am confident in my time management skills	3.6	4.1	0.206	3.81	4.286	**0.012**
I think of myself as a scientist	3.1	4	**0.038**	3.19	4.143	**0.002**
I think a career as a scientist includes participating in the broader scientific community	3.6	4.5	**0.038**	4.476	4.667	0.206
I think a career as a scientist includes working face to face with others	4	4.5	0.102	4.476	4.524	0.85
I think a career as a scientist includes providing direct help to others	4.1	4.5	0.18	4.333	4.476	0.257
I think a career as a scientist includes helping society	4.5	4.5	1.0	4.857	4.714	0.18
I see myself as a scientist	3.6	3.8	0.705	3.381	4.238	**0.003**
Science is important to me	3.6	3.9	0.317	4.667	4.714	0.739
It is important to me that others see me as a scientist	2.9	3.1	0.317	3.619	3.81	0.464

**Table 4.** Pre- and Post-Survey Data on Science Career Interest.

	Early High School Program			Late High School Program		
	Pre-survey Mean	Post-survey Mean	P-value	Pre-survey mean	Post-survey mean	P-value
How interested are you in pursuing the following:						
1. Research opportunities in HS	3.1	3.1	0.317	3.9	3.9	0.564
2. Undergraduate degree in a scientific field	2.9	3	0.655	3.8	3.7	0.317
3. PhD	3.0	3.3	0.783	3.2	3.1	1.000
4. Master’s degree	3.1	3	0.157	3.1	3.2	0.366
5. MD	2.6	2.9	0.705	3.2	3.4	0.083
6. Public health career	2.6	2.6	1	2.8	2.7	0.808
7. Career in scientific research	2.5	3.1	0.18	3.4	3.6	0.414

**Table 5.** Illustrative quotes from participants in the early high school science program.

Themes	Illustrative Quotes
COVID Impact / Virtual Programming	“I feel like being virtual like you always have time, like you, you weren’t doing stuff so you like always had time to like meet up with your group separately to work on like individual projects.” “I feel like the social distancing and being at home and not like, in person, made a big impact on how we met.. .like our experience and talking, having conversations and stuff like that, I feel like that’s like what would have been better if we did [the program] in person.” Interviewer: “Would you have preferred [virtual programming]?” Student: I think I would have, yeah Interviewer: Was there any benefit to having it virtually? Student: We got to meet new people from really far away “I think [the program] distracted me from like what’s really going on with this COVID stuff and having like [to] think about it, it distracted me from that and I have something to focus on.” “I would have to say that COVID like since I’m at home, like it’s boring to get on Zoom calls but like this specific Zoom call would be like so fun and like I talked to people and like other Zoom calls I wouldn’t talk…”
Career / Future Directions	“Like in the beginning, I like I did not want to get into the medical field, I was not interested at all, or anything like that. But then after the program I really want to start looking into like being a surgeon, because that seems very interesting to me now.” “I feel like no matter what I do I like I really want to have like a career in medicine.” “A lack of it (people in science careers), I think, so not a lot of people from my town actually go out and well, at least I believe not a lot of people come out of this town and kind of achieve great things, but I have heard some stories, but like I said it’s only some I kind of want to be part of that some to kind of add to those stories.”
Science Identity / Science Knowledge	“‘[I feel like a scientist] because I was able to like hear people’s opinions about depression and get data, statistics and I put a whole presentation together. It like felt like I was actually doing something.” “I thought that scientists we’re just like you know mixing bottles and you know, making medicine, so I realized there’s a lot of types of scientists and a lot of types of other careers that don’t necessarily have scientists in the name, but I think I would consider them as scientists.” “The beginning when like the word scientists came up to me, I would always think about somebody like a scientist as basically like somebody who like studies stuff as somebody who does not contribute anything they just say basically get information. I did not know, like they did stuff to come up with that information and like the whole process to get that information. I did not know any of that”
Diversity and Mentorship	“I never really like spoken to someone that was a lawyer, I don’t know anybody that’s a lawyer so like just hearing her story like when she was younger and. how she got into law school and all that and, like how she’s like a professor there it like inspired me because, like, I really wanted to be a lawyer at one point to like just hearing how she became a lawyer was like kind of cool to me.” “I mean, I know that they chose a lot of people, specifically because they were like black and brown folks and that was awesome because all of my girls were African American and I just love it when they, you know, can see someone that looks like them”
Program Design and Feedback	“The experience was really exciting and nerve wracking at the same time. um we had to work together, and it was like clashing heads, sometimes, and some people didn’t want to do it, at certain times so that was hard too. But at the end, it was really good and really exciting.” “So the kickstarter program it was one time a week instead of doing that I would want to do two times a week because sometimes I would forget that I even had a project to do and I will let time like slip through my fingers to a point where we were almost rushing at the end.”

**Table 6.** Qualitative written feedback from late high school student participants.

What students enjoyed about the program	“STEP-UP showed me that scientific research is not only confined to the lab, but also can be used as a way to influence the society we live in.” “Prior to this I viewed a career as a scientist as fictional and something that is made up in movies.” “I like the sessions on how to write an abstract and presenting the research.”
What students would change about the program	“This might be due to the virtual setting of this year’s STEP-UP program, but I do believe that the interaction between the interns of the <<blinded>> cohort was limited.” “I felt there wasn’t enough time to socialize casually with the other cohort members, but it could have also been because of the virtual environment.”

## ABBREVIATIONS

STEM: Science, Technology, Engineering, and Math
STEP-UP: Short-Term Research Experience Program to Unlock Potential
